# Are patients with COPD treated with NIV in accordance with national guidelines? An internal audit

**DOI:** 10.3402/ecrj.v1.24506

**Published:** 2014-11-11

**Authors:** Ingrid L. Titlestad, Fanny Olsen, Hanna M. Sandqvist, Melvin M. Pourbazargan, Håvard H. Fretheim, Annmarie T. Lassen, Jørgen Vestbo

**Affiliations:** 1 Department of Respiratory Medicine, Odense University Hospital, University of Southern Denmark, Odense, Denmark; 2 Department of Emergency Medicine, Odense University Hospital, University of Southern Denmark, Odense, Denmark; 3 Respiratory Research Group, Manchester Academic Health Science Centre, University Hospital South Manchester NHS Foundation Trust, University of Manchester, Manchester, UK

**Keywords:** non-invasive ventilation, respiratory failure, COPD, hypercapnea

## Abstract

**Introduction:**

Non-invasive ventilation (NIV) as an add-on modality to medical treatment has been recommended in national guidelines for patients acutely admitted with chronic obstructive pulmonary disorder (COPD) exacerbation and hypercapnic respiratory failure. To address concerns regarding whether NIV is used appropriately, we conducted an audit of COPD patients admitted to a university hospital in Denmark.

**Material and methods:**

Data from medical records were retrieved for two cohorts in 2010: 1) all patients admitted to the Medical Emergency Ward with the diagnosis of COPD, and 2) all patients receiving NIV regardless of their diagnosis at the Respiratory Ward. Demographic data and outcome of treatment were registered.

**Results:**

Cohort 1 comprised 804 admissions fulfilling criteria for COPD at evaluation, and of the 804 admissions, NIV was initiated in 151 (18.7%) admissions. In 42 additional cases (5.2%), initial mild respiratory acidosis was registered at admission, fulfilling criteria for NIV treatment; and, in 36 cases, the clinical status was reported as improved or not reported at all; no deaths were observed. In cohort 2, 124 admissions were registered that comprised 110 admissions with COPD and 14 without a diagnosis of COPD (of which half had a ‘not-to-intubate’ order). The indication for NIV treatment was met in 92.7% of the COPD admissions.

**Conclusion:**

NIV was initiated in 18.8% of the COPD admissions, and in an additional 5.2%, NIV criteria were met without initiation. In 82.3% of the admissions receiving NIV, a COPD diagnosis and correct criteria for NIV treatment were met.

Chronic obstructive pulmonary disease (COPD) is considered a global health issue (1). In Denmark, an estimated 400,000 subjects have COPD (2), and COPD is annually responsible for more than 20,000 acute hospital admissions (3).

Non-invasive ventilation (NIV) as an add-on modality to medical treatment is recommended in national guidelines for patients acutely admitted with COPD exacerbation and hypercapnic respiratory failure (4). This recommendation is based on randomized controlled trials showing lowered mortality rates in highly selected patients (5) and subsequent expert interpretation (6). A nationwide COPD quality improvement program – DrKOL, formerly NIP-KOL – was launched in Denmark in 2008, where data on the use of NIV and mortality have been monitored in patients acutely admitted with a COPD exacerbation. In this program, recently published data show regional variations in practice (7).

In the region of Southern Denmark, the ratio of acutely admitted COPD patients with exacerbation receiving NIV has been consistently higher than in other regions in Denmark throughout the years of registration (8). This practice has been partly explained by early implementation of NIV in the Respiratory and Medical Emergency Wards in the region, locally initiated targeted introductions to guidelines recommending this treatment modality, and consistent ICD-10 (International Statistical Classification of Diseases and Related Health Problems, 10th revision) coding practice (9, 10). In England, initial data on the mortality of COPD patients admitted with an exacerbation did not show improved survival after the publication of guidelines supporting the use of NIV, and a multicenter audit revealed that NIV was often given to patients outside indications, and that patients fulfilling criteria for treatment with NIV did not receive NIV treatment (11).

To address concerns on whether or not NIV is provided appropriately, we undertook an audit of all COPD patients admitted to a Medical Emergency Ward and a Respiratory Ward at Odense University Hospital, Denmark, in 2010. The aims of this study were 1) to evaluate if COPD patients with criteria for NIV therapy received the treatment according to local and national guidelines, and 2) if patients receiving NIV also fulfilled criteria of having COPD and respiratory acidosis.

## Methods

Two cohorts of patients admitted in 2010 were retrieved:

1. All patients admitted to the Medical Emergency Ward at Odense University Hospital with a diagnosis of COPD using the ICD-10 codes: COPD (DJ44) as a primary diagnosis, or respiratory failure (DJ96) or pneumonia (DJ13–DJ18) as a primary diagnosis in combination with COPD (DJ44) as a secondary diagnosis.
2. All patients retrieving NIV at the Respiratory Ward at Odense University Hospital, regardless of the ICD-10 codes used, and registered prospectively.

Data were retrospectively retrieved from electronic medical records. Baseline data registered were age, gender, and, from spirometry: forced expiratory volume in 1 second (FEV_1_), FEV_1_ in percentage of the predicted value (FEV_1_ %), and the FEV_1_/forced vital capacity (FVC) ratio. Diagnosis of COPD and use of long-term oxygen therapy (LTOT) were recorded, and comorbidities were registered when reported in the electronic medical records or if medical treatment was provided. Listed comorbidities were asthma, ischemic heart disease, hypertension, heart failure, atherosclerosis, stroke, malignancy, diabetes, osteoporosis, liver disease, rheumatoid arthritis, and chronic kidney failure. A ‘not-to-intubate’ order was registered when listed. Patients were monitored with repeated arterial analyses, and data captured when reported in the records.

### Organization of ward

Patients were admitted to the Medical Emergency Ward from either the general practitioner or the open Emergency Department at the hospital. Patients suspected of having an acute exacerbation of COPD based on clinical presentation, prior records, arterial blood gas analyses, and chest X-ray were assessed after initial standard treatment, administration of systemic steroids, and, if needed, antibiotics. Spirometry was not done routinely in the acute setting. Patients either continued standard medical treatment or were provided additional ventilator support with NIV in the Medical Emergency Ward. Criteria for NIV in COPD were: 1) arterial blood pH <7.35; and 2) PaCO_2_>6.0 kPa and PaO_2_<7.0 kPa, presenting with mono-organic symptoms of dyspnea and a respiratory rate>25. When possible, patients initiated on NIV were referred to the Respiratory Ward the next day. When NIV was initiated in the Medical Emergency Ward, standard NIV protocols were followed (initial inspiratory positive airway pressure 10 cm H_2_O and expiratory positive airway pressure 4 cm H_2_O), with the oxygen supplement aiming for a peripheral oxygen saturation measure of 90–92%. Changes in NIV pressure settings were made depending on the clinical situation and arterial blood gases, in accordance with a Danish National NIV Guideline (4). A specialist in respiratory medicine was available on call for consultation at all times.

## Results

In 2010, there were 825 admissions registered with an exacerbation of COPD according to the DrKOL registry. When evaluating the medical records, we found 21 patients who did not fulfill lung function criteria for COPD, resulting in 804 admissions of 521 unique patients. Of the 521 patients, 368 patients were admitted once, 96 twice, 29 three times, 12 four times, 5 five times, 8 six times, 1 seven times, one 10 times, and one 19 times. Demographic data on all COPD patients and admissions are presented in Table 1. Admissions are presented in order to demonstrate that readmissions were more frequent in patients with the lower spirometry measures and use of LTOT. Current smoking was registered in 31.1% of the patients. Of the 521 patients, 71 (13.6%) had no comorbidities, 62 (11.9%) had one, 116 (22.3%) had two, and 272 (52.2%) had three or more listed in their medical records.

**Table 1:** Cohort 1: Demography of verified COPD patients and of all admissions with a COPD exacerbation to a Medical Emergency Ward in Denmark in 2010 with ICD-10 codes COPD (DJ44) as a primary diagnosis, or respiratory failure (DJ96) or pneumonia (DJ13–DJ18) as a primary diagnosis in combination with COPD (DJ44) as a secondary diagnosis. Numbers (%) are shown if not stated otherwise

	All patients with verified COPD	All admissions of COPD exacerbation
Patients (male/female)	521 (205/316)	804 (304/500)
Age (years)^a^	72.6 [38, 65, 81, 94]	71.7 [38, 64, 80, 94]
Documented spirometry	383 (73.5%)	635 (79.0%)
FEV_1_ % of predicted^a^	37.8 [12, 26, 47, 101]	35.4 [12, 24, 43, 101]
Long-term oxygen therapy	59 (11.3%)	150 (18.6%)
Comorbidities		
History of asthma	39 (7.5%)	56 (7.0%)
Osteoporosis	141 (27.1%)	248 (30.8%)
Heart failure	93 (17.9%)	156 (19.4%)
Ischemic heart disease	103 (19.8%)	174 (21.6%)
Chronic kidney disease	24 (4.6%)	35 (4.4%)
Diabetes mellitus	79 (15.2%)	122 (15.2%)
Cerebral stroke	88 (16.9%)	132 (16.4%)
General atherosclerosis	42 (8.1%)	61 (7.6%)
Malignancy	62 (11.9%)	84 (10.4%)
Rheumatoid arthritis	23 (4.4%)	37 (4.6%)
Hypertension	131 (25.1%)	219 (27.2%)
Liver disease	23 (4.4%)	56 (7.0%)

a [minimum, 25th-percentile; maximum, 75th-percentile]. FEV_1_, forced expiratory volume in 1 second.

In this acute cohort, NIV treatment was initiated in 151 (18.7%) of the 804 admissions. Evaluation of admissions revealed 42 additional admissions (5.2%) in which arterial blood gas analyses fulfilled criteria for NIV. In most admissions (36), initial arterial blood gas analysis showed respiratory acidosis, but later analyses or evaluations of clinical states were either described as improved (28) or not commented on at all (8) in the patient records. No deaths occurred within the registered 30 days following these 36 admissions. In four admissions, NIV treatment was not initiated due to patients’ inability to cooperate or refusal, and two of these patients subsequently died, one patient within three months and the other 2.5 years later. In the last two cases, NIV treatment was considered futile, and both patients subsequently died in hospital. In the group of 151 patients in whom NIV was initiated, treatment in nine patients was abandoned quickly due to a ‘not-to intubate’ order or later refusal. These admissions have been categorized as adequate treatment choices. Thus, 157 admissions (81.3%) out of 193 admissions fulfilling the indication for NIV were correctly evaluated, and 151 admissions (78.2%) were subsequently treated with NIV acutely.

In the second cohort, which comprised patients receiving NIV during an admission to the Respiratory Ward in 2010, 124 NIV treatments in 105 unique patients were registered. A total of 91 patients received NIV once, 10 twice, 3 three times, and 1 four times. NIV was initiated with registered blood gas analyses pH<7.35 and pCO_2_>6.0 kPa in 104 cases (83.9%), and in 21 cases (19.3%) drowsiness was noted.

In this cohort, 93 treatments were initiated in patients also registered in DrKOL with ICD-10 codes, as described in this article. Fourteen treatments were initiated in patients who were subsequently discharged without a diagnosis of COPD, and in the remaining 17 admissions, COPD patients either were registered at the Respiratory Ward when being admitted directly to the Intensive Care Unit (ICU) or had another primary diagnosis than COPD (DJ44), pneumonia (DJ13–DJ18), or respiratory failure (DJ96). Demographic data presented in Table 2 as patients and admissions with a diagnosis of COPD compared to patients without a diagnosis of COPD. All patients registered without COPD and treated with NIV had only a single admission in the Respiratory Ward.

**Table 2:** Cohort 2: Characteristics of 105 patients and 124 admissions in which NIV was initiated, divided according to the presence or absence of a diagnosis of COPD. Numbers (%) are shown if not stated otherwise. There were no re-admissions of patients receiving NIV without COPD

	COPD patients	COPD admissions	Admissions (patients) without COPD
Patients (male/female)	91 (33/58)	110 (39/71)	14 (7/7)
Age (years)^a^	72 [39, 64.5, 74, 91]	72 [39, 64, 79, 91]	82 [46, 64, 84, 92]
Documented spirometry	81 (89%)	100 (91%)	7 (50%)
FEV_1_ % of predicted^a^	31 [10, 21, 38, 87]	29 [10, 21, 35, 87]	41 [17, 33, 50, 67]
Long-term oxygen therapy	25 (27.5%)	31 (21.2%)	2 (14.3%)
Comorbidities			
History of asthma	6 (6.6%)	6 (5.5%)	1 (7.1%)
Osteoporosis	27 (29.7%)	31 (28.3%)	1 (7.1%)
Heart failure	35 (38.5%)	44 (40.0%)	6 (42.9%)
Ischemic heart disease	17 (18.7%)	21 (19.1%)	0 (0%)
Chronic kidney disease	6 (6.6%)	7 (6.4%)	2 (14.3%)
Diabetes mellitus	21 (23.1%)	28 (25.5%)	4 (28.6%)
Cerebral stroke	15 (16.5%)	17 (15.5%)	2 (14.3%)
General atherosclerosis	6 (6.6%)	6 (5.5%)	1 (7.1%)
Malignancy	18 (19.8%)	22 (20.0%)	3 (21.4%)
Rheumatoid arthritis	2 (2.2%)	3 (2.7%)	0 (0%)
Hypertension	20 (22.0%)	21 (19.1%)	0 (0%)
Liver disease	3 (3.3%)	5 (4.5%)	0 (0%)

a [minimum, 25th-percentile; maximum, 75th-percentile]. FEV_1_, forced expiratory volume in 1 second.

In four admissions, NIV was given in the absence of hypercapnea (pCO_2_<6.0 kPa), and NIV treatment of all of these patients was categorized as inappropriate. Two of these patients treated had a ‘not-to-intubate’ order due to comorbidities (disseminated cancer and multimorbidity), and NIV was given as a last option; the other two were subsequently transferred to the ICU and intubated, and one patient died. The three patients without diagnosed cancer were elderly (79, 82, and 83 years), and only the patient with COPD survived.

Thus, for 103 (82.3%) of the 124 NIV treatments initiated, an indication of respiratory failure in COPD was documented.

The aim of this study has primarily been focused on the use of NIV treatment. Outcome data for COPD revealed treatment failure, defined as referral to the ICU, intubation, or death in patients presenting with an initial pH at 7.30 to 7.35 as 15% (3 of 20 patients), pH lower than 7.30 to 7.25 as 21.1% (8 of 38 patients), and pH lower than 7.25 with treatment failure in 36.7% (11 of 30 patients).

## Discussion

We present data on clinical practice and adherence to guidelines on NIV treatment to COPD patients presenting with exacerbation, and we found that 18.7% of patients admitted had received NIV treatment. A group of patients initially presenting with respiratory failure and mild acidosis (5.2%) were not treated with NIV, but in 36 of these 42 cases, an improved clinical status without supplementary ventilation support was documented. Few patients could not cooperate or refused treatment, but evaluation on whether these patients could have accepted NIV or benefitted from NIV treatment in another setting was not possible.

The decision regarding NIV treatment was considered for patients presenting with respiratory failure and hypercapnea, and NIV was given in 14 patients without verified COPD, where the treatment was offered as the best option in over half of the cases having an order of ‘not-to-intubate’. In four admissions, NIV treatment was initiated without hypercapnea, and the outcome for these admissions was categorized as ‘failure’ (referral to ICU or mortality). Confalonieri et al. (12) showed in a randomized trial in an ICU setting that NIV given to patients presenting with acute respiratory failure due to community-acquired pneumonia was superior to standard treatment (oxygen delivery in Venturi masks). Subgroup analysis showed that patients without COPD had lower measured pCO_2_ levels (4.27 kPa versus 9.73 kPa in the COPD group), and no significant reduction of intubation need was found.

Although our cohorts comprised patients with considerable comorbidities, our reported treatment failure, defined as transfer to the ICU (including treatment with NIV) or mortality, was comparable to the reported outcome in a randomized trial with selected COPD patients (13). Patients in our study with a pH level between 7.30 and 7.25 had a failure rate of 21.1%, and in the subgroup of patients with pH between 7.25 and 7.30 receiving NIV in the randomized trial, a failure rate of 36% was reported. Plant et al. showed that NIV treatment compared to standard treatment resulted in a significantly lower failure rate of 15% (NIV group) versus 27% (Standard), *p*=0.02, and our data from a real-life setting support that the intervention with NIV reduces intubation need, and thus referral to ICU, in COPD patients presenting with hypercapnic respiratory failure. In our cohort, 30 patients were registered with an initial pH lower than 7.25; treatment failure was seen in 11 of these 30 patients (36.7%), and five of them were referred to the ICU. Failure rates and mortality rates were comparable to those of the randomized controlled trial in the COPD group with a pH lower than 7.30 at presentation, and we have therefore concluded that initiation of NIV to COPD patients with a pH lower than 7.25 is feasible and safe in the Medical Emergency Ward and Respiratory Ward.

Our cohorts were primarily chosen firstly to try to include all COPD patients admitted to the Medical Emergency Ward and evaluate ICD-10 coding in clinical practice, and, secondly, to include all patients receiving NIV regardless of ICD-10. Corral-Gudino et al. (14) reported on a cohort of elderly COPD or cardiogenic pulmonary edema patients in Spain who had a ‘not-to-intubate’ order, and they concluded that use of NIV in general wards could be a safe and effective option, as a last-choice treatment. Not surprisingly, the outcome (survival at discharge) was better for COPD patients requiring NIV (63%) than for patients with acute cardiogenic pulmonary edema (55%), and this was also seen in survival rates after 1 year (50 and 37%, respectively). We have earlier reported long-term survival for COPD patients receiving NIV for the first time (10), and we found a 30-day mortality rate of 24.3%, but surprisingly a 5-year survival rate of 23.1%.

## Conclusion

NIV is implemented at the Medical Emergency Ward and Respiratory Ward as an add-on treatment in patients presenting with hypercapnic respiratory failure due to a COPD exacerbation. NIV was initiated in 18.8% of the COPD admissions, and in an additional 5.2%, NIV criteria were met without initiation, and the results are considered appropriate. In 82.3% of the admissions in the Respiratory Ward receiving NIV, a COPD diagnosis and fulfilled criteria for NIV treatment were met. In 11.2% of the admissions, patients did not have COPD; and, in half, NIV was also used as a last-choice treatment in patients with respiratory failure.
